# First Assessment of the Sex Ratio for an East Pacific Green Sea Turtle Foraging Aggregation: Validation and Application of a Testosterone ELISA

**DOI:** 10.1371/journal.pone.0138861

**Published:** 2015-10-14

**Authors:** Camryn D. Allen, Michelle N. Robbins, Tomoharu Eguchi, David W. Owens, Anne B. Meylan, Peter A. Meylan, Nicholas M. Kellar, Jeffrey A. Schwenter, Hendrik H. Nollens, Robin A. LeRoux, Peter H. Dutton, Jeffrey A. Seminoff

**Affiliations:** 1 Marine Mammal and Turtle Division, Southwest Fisheries Science Center, National Marine Fisheries Service, National Oceanic and Atmospheric Administration, La Jolla, California, United States of America; 2 Grice Marine Lab, University of Charleston South Carolina, Charleston, South Carolina, United States of America; 3 Florida Fish and Wildlife Conservation Commission, Fish and Wildlife Research Institute, St. Petersburg, Florida, United States of America; 4 Natural Sciences, Eckerd College, St. Petersburg, Florida, United States of America; 5 Veterinary Services, SeaWorld San Diego, San Diego, California, United States of America; Sonoma State University, UNITED STATES

## Abstract

Determining sex ratios of endangered populations is important for wildlife management, particularly species subject to sex-specific threats or that exhibit temperature-dependent sex determination. Sea turtle sex is determined by incubation temperature and individuals lack external sex-based traits until sexual maturity. Previous research utilized serum/plasma testosterone radioimmunoassays (RIA) to determine sex in immature/juvenile sea turtles. However, there has been a growing application of enzyme-linked immunosorbent assay (ELISA) for wildlife endocrinology studies, but no study on sea turtles has compared the results of ELISA and RIA. This study provides the first sex ratio for a threatened East Pacific green sea turtle (*Chelonia mydas*) foraging aggregation, a critical step for future management of this species. Here, we validate a testosterone ELISA and compare results between RIA and ELISA of duplicate samples. The ELISA demonstrated excellent correspondence with the RIA for providing testosterone concentrations for sex determination. Neither assay proved reliable for predicting the sex of reproductively active females with increased testosterone production. We then applied ELISA to examine the sex ratio of 69 green turtles foraging in San Diego Bay, California. Of 45 immature turtles sampled, sex could not be determined for three turtles because testosterone concentrations fell between the ranges for either sex (females: 4.1–113.1 pg/mL, males: 198.4–2,613.0 pg/mL) and these turtles were not subsequently recaptured to enable sex determination; using a Bayesian model to predict probabilities of turtle sex we predicted all three ‘unknowns’ were female (> 0.86). Additionally, the model assigned all turtles with their correct sex (if determined at recapture) with 100% accuracy. Results indicated a female bias (2.83F:1M) among all turtles in the aggregation; when focusing only on putative immature turtles the sex ratio was 3.5F:1M. With appropriate validation, ELISA sexing could be applied to other sea turtle species, and serve as a crucial conservation tool.

## Introduction

Understanding the demography of wildlife populations threatened with extinction is essential for developing sound conservation and management plans [1]. Population abundance, survivorship, and sex ratio are among the most fundamental demographic parameters, and these factors provide a context for monitoring population trends and modeling population viability over long time frames [2]. For example, information on the proportion of females in a population can shed light on its reproductive potential. When examined at multiple sites across broad geographic regions and diverse habitats, evaluations of sex ratio can help illustrate sex-related habitat preferences and perhaps identify sex-biased threats that impact a population. Indeed, information on sex ratios is vital for population assessment and management, but can be elusive with species and life stages that do not exhibit external sex-specific characteristics (e.g. seabirds, cetaceans, and sharks; [3–5]).

The sex of sea turtles is not distinguishable externally in immature stages. Sex can be distinguished for adult male sea turtles because they have long and muscular tails compared to females [6]. However, using short tail length to determine the sex of adult foraging females has been problematic at times because some turtles of adult size [e.g. as large as 90.5 cm curved carapace length (CCL)] with short tails are actually prepubescent male turtles incorrectly identified as female [7–10]. Therefore, one should be cautious when using external characteristics to determine sex in sea turtles. Furthermore, using molecular tools to determine sea turtle sex is inherently difficult because sex is not genetically determined in sea turtles [9]. Instead, sea turtles have temperature-dependent sex determination where the sex of a developing sea turtle embryo is influenced by the surrounding environment [11]. Sex is determined during the middle third of incubation (the thermosensitive period) during which cooler temperatures produce males and warmer incubation temperatures produce more females [12–14]. If incubated at a constant temperature, there is a pivotal temperature that will produce a 1:1 sex ratio and this can vary within and between species (27.7 to 31.0°C, [15]), but variation on either side of that pivotal temperature has the potential to produce embryos of a single sex. Knowledge of sex ratios has practical application considering that some conservation practices (e.g. hatcheries and nest relocation) may alter the thermal environment of sea turtle embryos with unintended consequences (i.e. [16,17]). In addition, information on sex ratios obtained over long time frames can help decipher the impacts of global climate change on populations [18].

Efforts to track changes in sea turtle sex ratios often focus on nesting beaches, where the sex of hatchlings (i.e. primary sex ratio) can be inferred from nest sand temperature or direct examination of gonadal histology from expired or sacrificed hatchlings [14,19]. However, because of low survivorship after hatching, the extent to which hatchling sex ratio is conserved in immature and adult life stages is unclear. Thus, characterizing sex ratio at foraging areas, where a larger cross-section of life stages is present, can offer greater insight into the functional sex ratio of a population [20].

There are multiple methods to determine the sex of immature turtles [6], including laparoscopy [21], histological examination of gonads from live or dead stranded turtles [22], or hormonal assay for testosterone (T) concentration in blood plasma [23]. However, all of these methods have deleterious impacts to the turtles or are logistically challenging, except blood collection for hormone assay. More specifically, expert training is required to perform the laparoscopy and it is an invasive technique that can be challenging to perform in the field. Histological examination generally requires sacrifice of the animal to obtain the gonadal sample or proficiency in necropsy and gonadal identification. In addition, stranded turtles may not represent an unbiased sample. Therefore, many studies collect blood samples because this technique can be minimally invasive and can be used to determine T concentration, and thus sex. The ease with which blood can be collected allows for adequate and representative sample sizes to make inferences about a population or aggregation.

The range of T concentrations for male and female sea turtles can vary between species and even between foraging aggregations within a species [6,24]. It is, therefore, important to determine the T range for individuals from each foraging aggregation and each species. Additionally, some studies using T to determine sex ratio of immature turtles have identified a T concentration range associated with uncertainty—i.e. the sex cannot be determined in these turtles because T concentrations fall between male and female ranges or there is overlap in T concentrations between males and females (e.g. loggerhead and green turtles, [25–27]). Some immature individuals will have T concentrations which fall within the range of male-female overlap, either by chance or possibly due to time of year/water temperature (see [28] for immature loggerheads), while for other populations/species the T concentration range for each sex is more defined (e.g. hawksbills, [29]). Adult-sized turtles can be misidentified as one sex based on T concentrations (i.e. reproductively active female with high T concentrations may be considered an immature or mature male) and hormone assays alone should not be used to determine the sex of turtles near the size threshold for maturity without additional information to corroborate the assay results. Morphological data on carapace size and tail length of sea turtles, as well as information on reproductive status and size at sexual maturity, greatly enhance the interpretation of the results of sex ratio studies. This limitation highlights the need to determine this ‘unknown’ range for each new foraging aggregation and species studied and is integral for studies using T to determine immature sea turtle sex ratios. However, the unknown range can be better defined with greater sample sizes and validation of sex determined by T concentration with known sex via laparoscopy.

Circulating T levels in sea turtles have typically been measured by radioimmunoassay (RIA, reviewed by [30]). However, enzyme-linked immunosorbent assay (ELISA) is preferable over RIA because it does not require radioactive reagents [31]; ELISA use is becoming more widespread due to lower set-up cost and because the smaller-sized ELISA plate readers are also portable enough for field use when compared to the large-sized gamma and scintillation counters used for RIA analysis. Enzyme-linked immunosorbent assay has been applied in marine wildlife to monitor health, reproduction, and stress response and to determine sex (e.g. fish, marine mammals, and sea turtles [27,32–35]). However, assay methods must first be validated to confirm reliability with each target species prior to application of a novel hormone assay technology. While this can be accomplished by comparing the results of such tests with internal gonadal examination via laparoscopy [6], a more practical solution is to compare sex determinations from new assay systems with results from a validated assay (e.g. RIA, [9,25]) on duplicate samples. To date, however, no sea turtle endocrinology study has yet determined consistencies between the antibodies used in a commercially available ELISA and the different antibodies of a customarily-used RIA as has been done for other wildlife species (e.g. big cats, [36]).

Despite the acknowledged conservation need for increased information on demography of sea turtles [37,38], sex ratio in foraging habitats has not been examined sufficiently [37]. Here we present findings from three projects: (1) validation of a T ELISA for use with green turtle (*Chelonia mydas*) plasma by comparing results obtained from RIA and ELISA techniques for turtles whose sex was known by laparoscopy, (2) application of the ELISA to a temperate foraging aggregation of green sea turtles in the eastern Pacific Ocean to determine sex ratio, and (3) development of a statistical model to estimate the probabilities of assigned sex for immature turtles which fall within 'unknown' ranges of T assay concentrations. To our knowledge, this is the first study to use hormone assessment techniques to study sex ratio for a sea turtle population in the eastern Pacific Ocean, despite numerous studies on sea turtle biology in the region (e.g. [39–41]).

The foraging aggregation of green turtles in San Diego Bay (SDB) has been studied for ~25 years and the congregation of turtles near power plant warm effluent water allowed for easy access to individuals (e.g. [42–47]). The aggregation (mean: 37.2 and range: 6–61 individuals, [42]) has been continually monitored and extensively sampled and offers a valuable opportunity to test the application of the ELISA on green turtles in the wild. This foraging aggregation of turtles is comprised of ‘black’ green turtles (*Chelonia mydas agassizii*) that are part of the threatened East Pacific population [48] and nest primarily in three locations in Mexico (Michoacán, Revillagigedo Archipelago, and Tres Marias Islands, [44]). Nesting occurs almost year-round but peaks in Oct/Nov in Michoacán and in the Revillagigedo Archipelago [49,50].

This study takes advantage of and builds upon a rich set of mark-recapture studies and research projects on a broad range of topics for this aggregation. These data will serve as a baseline for future assessments of sex ratio at this foraging habitat and also provide for comparisons with other studies in this region. Further, we believe validation of the ELISA technique and eventual real-world application to all sea turtle species will increase the speed and ease of hormone analysis for sea turtles. In addition, this study uses physiological tools to answer important ecological questions when traditional ecological metrics cannot be used.

## Methods

### Ethics Statement

Samples collected from captive sea turtles at SeaWorld were obtained as part of the routine veterinary care and are, therefore, exempt from requiring IACUC approval. In addition, the sea turtles housed at SeaWorld were obtained prior to the U.S. Endangered Species Act so these turtles and their offspring do not require a display permit.

The plasma samples of laparoscoped green turtles used to compare results of the ELISA and RIA assays were exported to the US under Panama CITES export permits INRENARE 2–94, ANAM SEX/A-077-04, ANAM SEX/A-56-07, ANAM SEX/A-73-08, ANAM SEX7A-056-09 and Bermuda CITES export permit 08BM0016. The US CITES import permit for both Panama and Bermuda is US758093/9. Research in Panama is conducted under Smithsonian Tropical Research Institute IACUC number 2014-0515-2017-2. IACUC is not required for research led by the Bermuda Turtle Project as it is conducted in collaboration with the Bermuda Aquarium, Museum and Zoo (BAMZ). All research on sea turtles in Bermuda water is required to be sanctioned by the Bermuda Government and is permitted at the discretion of the Director of the Department of Conservation Services; the BAMZ falls within the Department of Conservation Services. The BAMZ is accredited by the Association of Zoos and Aquarium and is mandated to have an Animal Welfare and Enrichment Committee as well as a Research and Conservation Committee. Any research on animals using invasive techniques involving BAMZ staff or resources requires the sanction of both committees.

The SDB research was conducted in accordance with the Animal Welfare Act, under Southwest Fisheries Science Center/Pacific Islands Fisheries Science Center IACUC (SWPI2013-04) policy, and permit approvals from the National Marine Fisheries Service (697, 988, 1297, 1591, 16803) and State of California Department of Fish and Game Permit (0166).

### ELISA Validation

#### Hormone Extraction

Steroid hormones were extracted from plasma following Wibbels et al. [26]. In brief, 50–500 μL of plasma was aliquoted into a glass tube (Catalog # 14-961-30, Fisher Scientific, Fair Lawn, NJ) and 4 mL of anhydrous ethyl ether (Fisher Scientific) was added to extract T from the plasma as T may be bound to proteins in the blood. Then, each glass tube (with plasma and ether) was placed in liquid nitrogen where the plasma layer was frozen and the ether layer (containing the hormones) was decanted into a new glass tube and dried under a steady stream of nitrogen gas (~20 min). Samples were reconstituted with 1.1 mL of 100% acetone (Fisher Scientific) in order to increase extraction efficiency (or recovery of T from the sample) by keeping it in concert with an organic solvent. Following acetone reconstitution, 1 mL aliquots were air-dried overnight (~16 hr). Extracted samples were reconstituted in 250 μL of 0.01 M phosphate buffered saline (PBS, Sigma, St. Louis, MO) with 0.1% bovine serum albumin (BSA, Amresco, Solon, OH), vortexed for 15 min, and then incubated in a water bath for 30 min.

Extraction efficiency was determined by adding (spiking) 10.0 ng of T from the ELISA T assay kit, which was used to make the standards, to a plasma sample of known low T concentration prior to extraction [51–53]. We extracted and quantified the amount of T in this spiked sample (diluted 1:100) as well as in an aliquot of the same sample of known low T concentration without added T (non-spiked). The ratio of the final T measurement minus the amount from the native non-spiked sample over the known quantity of T added was used to estimate extraction efficiency.

#### ELISA Testosterone Assay

A commercially available T ELISA kit (Catalog # ADI-900-065, ENZO Life Sciences, Plymouth, PA) was used to determine T concentration in each extracted plasma sample and all samples were quantified in duplicate. Standards (n = 5) of known T concentration (7.81–2,000 pg/mL) were prepared according to the assay kit protocol using PBS (with BSA) and, therefore, PBS (with BSA) was used as the ‘zero’ (B_0_) standard; however, we continued the serial dilution (1:2) of the standards to create two additional standards (3.905 and 1.9525 pg/mL) for a total of 7 standards in order to better characterize the 7.81 pg/mL standard, allowing the STD curve to have a full sigmoidal form. Although values within this low T concentration range have high variability, the two additional standards allowed us to obtain the T concentration of 5 plasma samples (out of 126 plasma samples) for which the T concentration would be undetectable (< 7.81 pg/mL) when simply using the standards suggested in the assay kit protocol. Due to the addition of the two lower standards, the sensitivity of the assay (2.0 pg/mL) was determined as the concentration of T measured at two standard deviations (SD) from the 0 (B_0_) standard along the standard curve when comparing multiple replicates (n = 6) of the 0 (B_0_), 1.9525, 3.905, and 7.81 pg/mL standards. The effective sensitivity of the assay is 1.2 pg/mL after correction for plasma volume, acetone volume, extraction efficiency, reconstitution volume, and dilution (see equation in data analysis section below). The T ELISA has 100% reactivity with testosterone, 14.64% reactivity with 19-hydroxytestosterone, 7.20% reactivity with androstenedione, 0.72% reactivity with dehydroepiandrosterone, 0.40% reactivity with estradiol, as well as <0.001% crossreactivity with estriol, corticosterone, cortisol, cortisone, estrone, progesterone, and pregenolone.

In order to evaluate assay drift, the standards and the same control sample (mean: 889.4 pg/mL) was used in each assay at the beginning and the end of each plate (see example plate layout, S1 Fig) and, as well, samples were assayed in random order. No detectable trend was observed in the values of the control samples with respect to time, indicating no significant drift.

All samples were assayed undiluted following reconstitution in PBS (with BSA). For samples that had T concentrations above the detection limit of the assay, we diluted (in PBS with BSA) a subsample of the plasma sample extract and obtained a reliable T concentration on a subsequent assay.

A Tecan spectrophotometer (Model: Sunrise, Phenix Research Products, Candler, NC, USA) was used to read the optical density within each well of the ELISA plate. The resulting T concentrations (pg/mL) were computed using a five-parameter logistic curve fitting program (Magellan 3.11, Tecan Group Ltd., Männedorf, Switzerland).

#### Assay Precision

Plasma samples from green turtles [7 immature males (determined by T concentration), 1 adult male and 5 adult females] housed at SeaWorld San Diego were used for extraction efficiency, quality control and parallelism validations for the assay. All plasma samples were collected at the discretion of the attending veterinarian as part of routine veterinary care. The mean ± SD straight carapace length (SCL) was 72.4 ± 14.6 cm (range: 54.6–97.6 cm). Straight carapace length was converted from CCL measurements using an equation [*SCL* = (*CCL* − 0.64)/1.06] for the San Diego Bay foraging population of East Pacific green turtles [44].

For quality control, an adult male green turtle plasma sample with known (determined by initial ELISA measurement) high T concentration (90,800 pg/mL) was extracted and T concentration of a diluted subsample was determined in each assay for this same sample to confirm both T within-assay (intra-assay) and between-assay (inter-assay) variation. We would re-run an entire assay (n = 0) if the T concentrations of the control sample ran in each assay did not fall within two SDs of the mean of previous values (mean ± SD: 889.4 ± 135.9 pg/mL).

The assay was validated for use with green turtle plasma by demonstrating parallelism, where the slopes of plotted curves from serial dilutions of the hormone standard provided in the ELISA kit were compared to serial dilutions of pooled plasma extracts (n = 4) of unknown T concentration. Serial dilutions of the standards and pooled plasma extracts were assayed in duplicate and triplicate, respectively.

We also did a matrix-interference test to examine if there was any potential interference caused by substances within our plasma samples, which are independent of specific antigen-antibody binding. We made a sample pool (n = 3) with low T concentration (8.2 pg/mL) and spiked aliquots (120 μL) of the pooled sample with an equal volume (120 μL) from each of the assay standards. The concentration of T contributed from the pooled sample was subtracted from each sample-spiked measurement so its contribution would be factored out of the assessment. A simple linear regression was used to determine the degree to which the measured T concentration corresponds to the true concentration of the spiked sample.

Within each assay run if the associated standards were not monotonic increasing values, we would discard the inaccurate duplicate of the standard from the curve (i.e., the value causing the deviation from monotonic progression); the resulting curve was deemed allowable if the curve fell within two SDs of the mean of remaining control values.

A sample would be re-assayed if the variation between duplicate analysis of the same sample was > 10% (n = 14). We also re-extracted and re-assayed samples where the T concentrations were anomalous (n = 16; e.g. a sample had unusually high or low T concentrations for that size/sex of turtle).

#### Data Analysis

Raw concentrations that were obtained from the ELISA analysis software were corrected for plasma volume, acetone volume, extraction efficiency, reconstitution volume, and dilution, using the equation: Testosterone (pg/mL)_ = ([Raw]*[Recontitution Volume (mL)])/([Plasma Volume (mL)]*([Acetone Volume (mL)]*[Extraction Efficiency]*[Dilution]). Data are presented as the mean ± standard error of mean (SEM) of the duplicate values for each sample.

To identify parallelism, we performed a correlation analysis to compare slopes of the log-transformed binding curve, specifically the central linear portion (~0.5 B/B_0_).

### RIA Testosterone Assay

RIA samples were extracted using previously published methodology described above [26], which was also used to extract plasma samples assayed via the ELISA. Extracted samples were reconstituted in 100 μL 0.05 M TRIS buffer (Sigma-Aldrich, St. Louis, MO) and T concentration was determined using a previously published and validated in-house RIA protocol ([23,26]; but see [28,54,55]). In brief, the RIA used for this study is a standard, widely applied single antibody, competitive-binding assay using tritium as the radiolabel and a charcoal separation step. Briefly, the protocol is as follows: (1) add antibody and radioactive T to each sample tube and incubate ~24 hr at 4°C (along with standards and B_0_), (2) add 1 mL charcoal solution (charcoal in TRIS serves to separate the bound and unbound T fractions in the sample) and let sit at 4°C for 15 min, (3) vortex at 4°C for 15 min (2300 rpm), (4) decant supernatant into scintillation vials and let sit overnight, and (5) run on scintillation counter the next day. All samples were quantified in duplicate. Seven standards of known radioinert T concentration (19.5–1,250 pg/100 μL) in TRIS buffer were used to generate a standard curve with TRIS buffer being used as a ‘zero’ (B_0_) standard. The sensitivity of the assay was approximately 10 pg/mL. Mean extraction efficiency was 92.9% and the mean intra-assay coefficients of variation was 9.7%; we did not calculate an inter-assay coefficient of variation due to samples being ran on a single assay. The antiserum used in the T RIA (T3-125, Esoterix, Calabasas, CA) has 100% reactivity with testosterone, ≤ 52% with delta-1-testosterone (cross-reactivity), 20% with dihydrotestosterone and delta-1-testosterone, 3.6% with 4-androsten-3b-17b-diol, 1.8% with 5a-androstan,3b,17b-diol, 0.5% with delta-4-androstenedione, 0.3% with 5b-androstan-3a,17b-diol, 0.14% with estradiol, 0.1% with estriol, 0.09% with 4-androsten-3,11-17-trione, 0.04% with deoxycorticosterone, 0.04% with progesterone, 0.02% with 5b-androstan-3,11,17-trione, as well as ≤ 0.01% with aldosterone, androsterone, coritcosterone, cortisol, cortisone, dehydroepiandrosterone, epiandrosterone, estrone, etiocholanolone, pregnanediol, pregnanetriol, 11-deoxycortisol, and 17a-hydroxyprogesterone.The resultant hormone concentration therefore denoted total androgens.

### ELISA and RIA Results Comparison

To validate the ELISA T assay against an already-validated RIA, we determined the T concentrations of 30 duplicate plasma samples (extracted following the same methodology described above) previously analyzed using RIA (S1 Table) and compared the results of the two assays using a correlation analysis. Plasma samples were obtained from known sex (via laparoscopy) wild-caught immature and adult green turtles [n = 30; mean ± SD (range), 58.2 ± 20.7 cm (28.5–104.6) cm SCL] captured in nets in developmental habitats (Bermuda and Panama) or along a migratory corridor (Caribbean Panama) as part of ongoing research programs. Blood samples were collected in sodium/heparin tubes [within 2 hr of capture for Bermuda sea turtles; in the case of Panama, blood samples for all but one animal were taken in < 17 hr (one at a maximum of 26 hr depending on when it was captured in the net]. Due to the longer time frame from capture to blood collection, we investigated the potential effects of capture/handling stress on circulating T concentrations and we found no significant differences in T concentrations 6 or 12 hr after baseline samples were collected upon capture; therefore, capture stress did not confound our ability to predict sex using T concentrations [56]. After blood collection, animals were laparoscoped to determine sex and maturity status, as well as to calibrate the RIA (methods described in [10]). Blood samples were centrifuged and plasma aliquots were frozen until assay; samples were initially stored frozen (-20°C, Bermuda samples; liquid nitrogen, Panama samples), then carried to the U.S. as baggage in thick-walled coolers chilled by frozen gel packs and were ultimately stored at -50°C until they were shipped on dry ice to the laboratory for hormone analysis. All samples were analyzed for hormone concentration soon after sampling (<1 month for Bermuda samples and < 1 yr for Panama samples) via RIA in 2008 and then again a few years later (2014) via ELISA (see S1 Table for sample collection dates). Information about the sex (via laparoscopy) of these turtles was unknown to the researchers performing either the RIA or ELISA assays.

#### Data Analysis

When comparing the results obtained from the ELISA and RIA, all T concentrations were log-transformed prior to statistical analysis in order to reduce heteroscedasticity (i.e., increasing T concentration produced an increase in measurement variance).

### ELISA Measurements of San Diego Bay Turtles

#### Study Site

**Fig 1:**
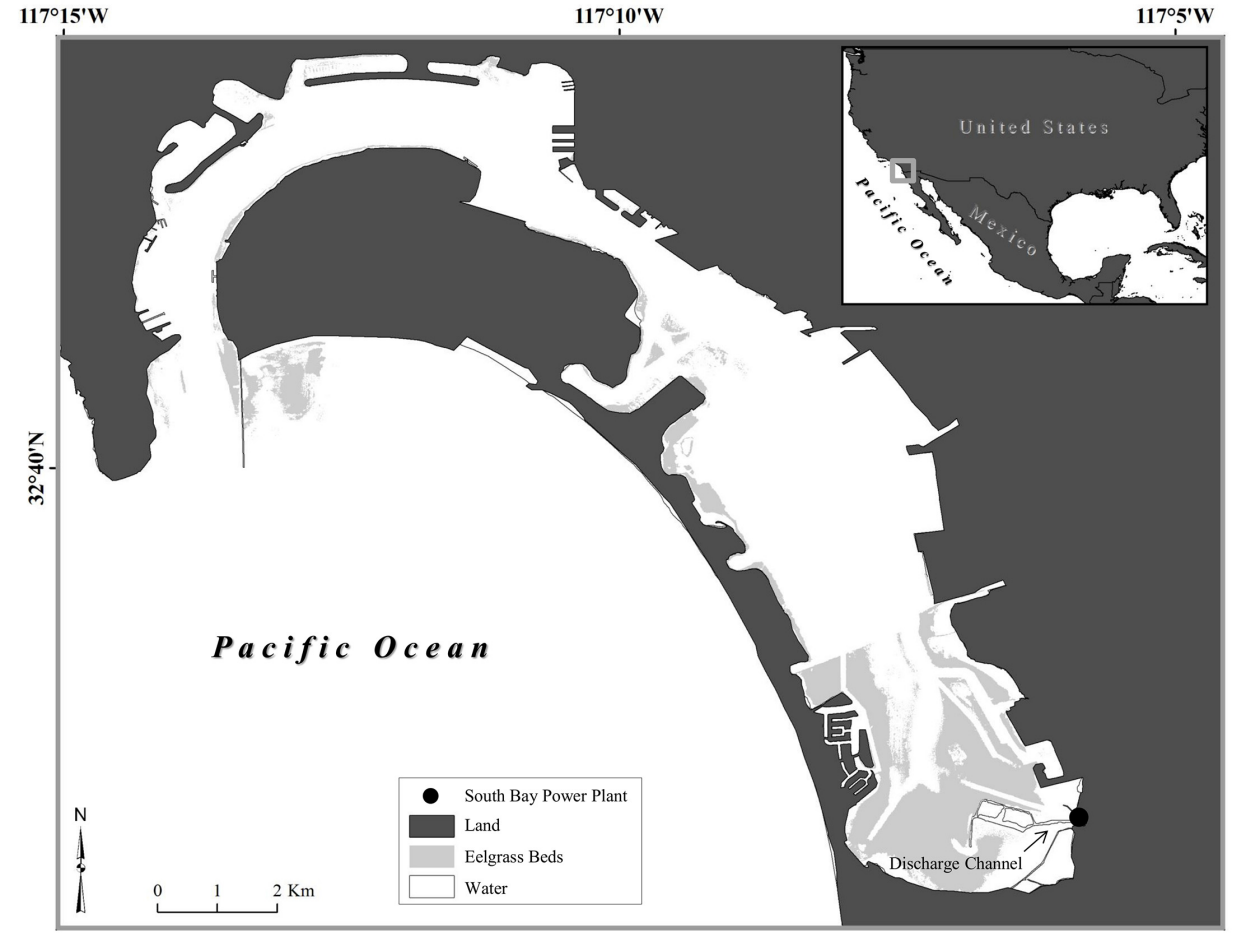
San Diego Bay foraging area of East Pacific green sea turtles.

San Diego Bay (SDB; Fig 1), a temperate foraging area for green turtles, is characterized by eelgrass (*Zostera marina*) beds, benthic marine algae, salt marshes, and invertebrate communities [57]. The northern part of this 25-km long bay is urbanized with heavy boat and shipping traffic, whereas in the south there is an ecological reserve [58] where the turtles spend the majority of their time [59]. Monthly average sea surface temperatures (SST) within SDB range from 12.8–18.3°C in the winter months and maximum monthly average SST in the summer months was from 22.2–26.4°C [60]. However, turtles were mostly captured in the discharge channel of the south bay power plant (Fig 1) where the monthly average SST was much warmer in winter (20.0–23.9°C) and the maximum monthly average SST was extremely warm during the summer (31.7°C) compared to the rest of the bay [60].

#### Turtle capture and measurement

Between 1990 and 2014, green turtles were captured with entanglement nets (100 m x 8 m, mesh size = 40–60 cm knot-to-knot) in the southern portion of San Diego Bay. Turtles were captured year-round but prior to 2011 capture-and-release efforts occurred more frequently during winter months (began in late autumn and ended in spring), a time period during which turtles congregated in the southern portion of the bay in and around warm water effluent from the power plant [46]. However, since the closure of the power plant in Dec. 2010, more intense capture efforts in the eelgrass beds surrounding the previous location of the power plant occurred during the months when SDB has warmer water temperatures (May-October) and when the turtles are more likely to be physically active. Straight carapace length was measured (± 0.1 cm) from the nuchal notch to the posterior-most portion of the rear marginal scutes using a forester’s caliper. Turtles were weighed to the nearest kg using a 500-kg electronic balance and tagged with Inconel tags (Style 681, National Band and Tag Company, Newport, Kentucky) in the first large proximal scale of one of the front flippers. We measured tail length (TL; ± 1.0 cm) from the tip of the tail to the trailing edge of the plastron using a flexible measuring tape. Turtles smaller than 90.0 cm SCL were considered immature (growth rates were zero or negative for turtles greater than this size, indicating adulthood, [44]). Individuals with tail length > 30 cm and SCL > 90.0 cm were considered putative adult males, and those with short tails and SCL > 90.0 cm were considered putative adult females. The mean (range) female size at nesting is 76.8 (56.0–95.6) cm SCL at Michoacán [61] and 88.5 (75.8–102.0) cm SCL at Revillagigedo Archipelago [49]. Straight carapace length was converted from CCL measurements using the previously described equation [44]. Genetic studies indicate that the SDB aggregation includes individuals from several Mexican breeding sites with more turtles likely originating from the Revillagigedo Archipelago than Michoacán ([62]). However, because male and female green sea turtles may mature at different sizes and over a range of sizes [10], we acknowledge that mature female turtles < 90 cm SCL would be incorrectly classified as immature. Nevertheless, because the primary goal of this study is to determine sex of immature turtles, using the relatively large 90 cm SCL delineation should prevent misidentification of large immature males as adult females. We collected blood samples from 69 individual green turtles in SDB, with multiple blood samples collected from 19 turtles that were captured on several occasions (n = 96 total samples, S2 Table) during different years or the same year (n = 1). Sex was determined for 39 turtles upon recapture based on SCL and tail length. The mean ± SD (range) SCL of the turtles in this study was 82.6 ± 17.1 cm (45.4–109.3 cm).

#### Blood collection and handling

We collected blood samples (maximum volume < 5 mL/kg) from the dorsal cervical sinus [61] using a 1.5 inch, 21 gauge vacutainer needle (Becton, Dickinson and Company, Franklin Lakes, NJ) and a 10 mL sodium heparin vacutainer blood collection tube (Becton, Dickinson and Company). Although blood collection immediately after capture is ideal to reduce the influence of capture stress on circulating hormone concentration, it was not always possible due to logistical constraints (large majority collected within one hour). Samples were kept chilled on ice packs until centrifugation (3000 x g for 10 min) after field work completion that day. Following centrifugation, plasma was aliquoted into 2 mL cryovials (Corning Inc., Corning, NY) and stored at -80°C (see S2 Table for date of collection) until the assays were conducted (in 2013 and 2014).

#### Estimating Probability of Sex Assignment

We developed a statistical model for the relationship between T and various covariates, including sex. The log-transformed and standardized observed T quantities in subsamples from the *c* ^*th*^ capture of the *i* ^*th*^ individual were treated as a random sample (n = 2) from a normal distribution with a mean (μ_i,c,j_) and variance (σ^2^), where the variance was treated as equal among all individuals. The mean (μ_i,c,j_) was assumed to be a linear function of covariates, including sex, size (SCL), day of capture (DOY), and tail length (TL). The full model included all covariates: μ_i,c,j_ = β_0_ + β_sex_ × (sex_i_) + β_SCL_ × (SCL_i,c_) + β_DOY_ × (DOY_i,c_) + β_TL_ × (TL_i,c_). We standardized (mean = 0 and SD = 1) all covariates, except sex. For combining the data from SDB (n = 69) and Bermuda/Panama (n = 30), we used common parameters for all but DOY, considering a possibility that seasons may affect the T concentrations differently between temperate and tropic foraging areas. In addition, it cannot be assumed that East Pacific and Atlantic green turtles have the same seasonal pattern of reproduction or age/size at maturity; samples from these two groups were used to demonstrate correlation between the results of the assays, as well as to ground-truth the sex determinations. Turtles with unknown sex were given missing values for the sex covariate and were predicted in the analysis. Specifically, the sex of each turtle [known sex via laparoscopy or tail length/T concentration at additional captures (Bermuda/Panama n = 30 and SDB n = 47) or unknown sex] was modeled as a Bernoulli trial with unknown probability [63,64]. Because of the lack of other variables that can inform the probability of sex but which were not already in the main model (e.g., tail lengths and T concentration), the probability of sex (expressed as the probability of being male) was assumed to be uniformly distributed between 0 and 1. We also considered the following models with fewer covariates: (1) Sex+DOY+SCL, (2) Sex+DOY, (3) Sex+SCL, (4) Sex+SCL+TL, (5) Sex+DOY+TL, and (6) Sex only. Performance of the models was compared using Deviance Information Criteria (DIC), a metric for model comparison in Bayesian statistics where smaller values indicate a better fit [65].

Posterior distributions of the parameters were obtained through Markov chain Monte Carlo (MCMC) sampling using JAGS language (v. 3.4.0, [66]) and executed in the R statistical environment (v.3.1.2, [67]) via the RJAGS package [68]. We ran five independent chains of 10,000 burn-in steps, followed by 20,000 steps to sample from the joint posterior distribution. The Gelman-Rubin convergence diagnostics statistic was used to determine the convergence of the chains. During the MCMC sampling, the number of times the samples were drawn from each sex was counted. The proportions then were treated as the probability of each sex. The appropriateness of the model to the observed data was determined via posterior predictive simulations, in which possible data were simulated using the random samples from the joint posterior distribution of the parameters. If the majority of observed data points were within the ranges of simulated data, the model was assumed to be appropriate [65]. JAGS and R code used in this analysis are available upon request.

## Results

### ELISA validation

#### Assay Precision

**Fig 2:**
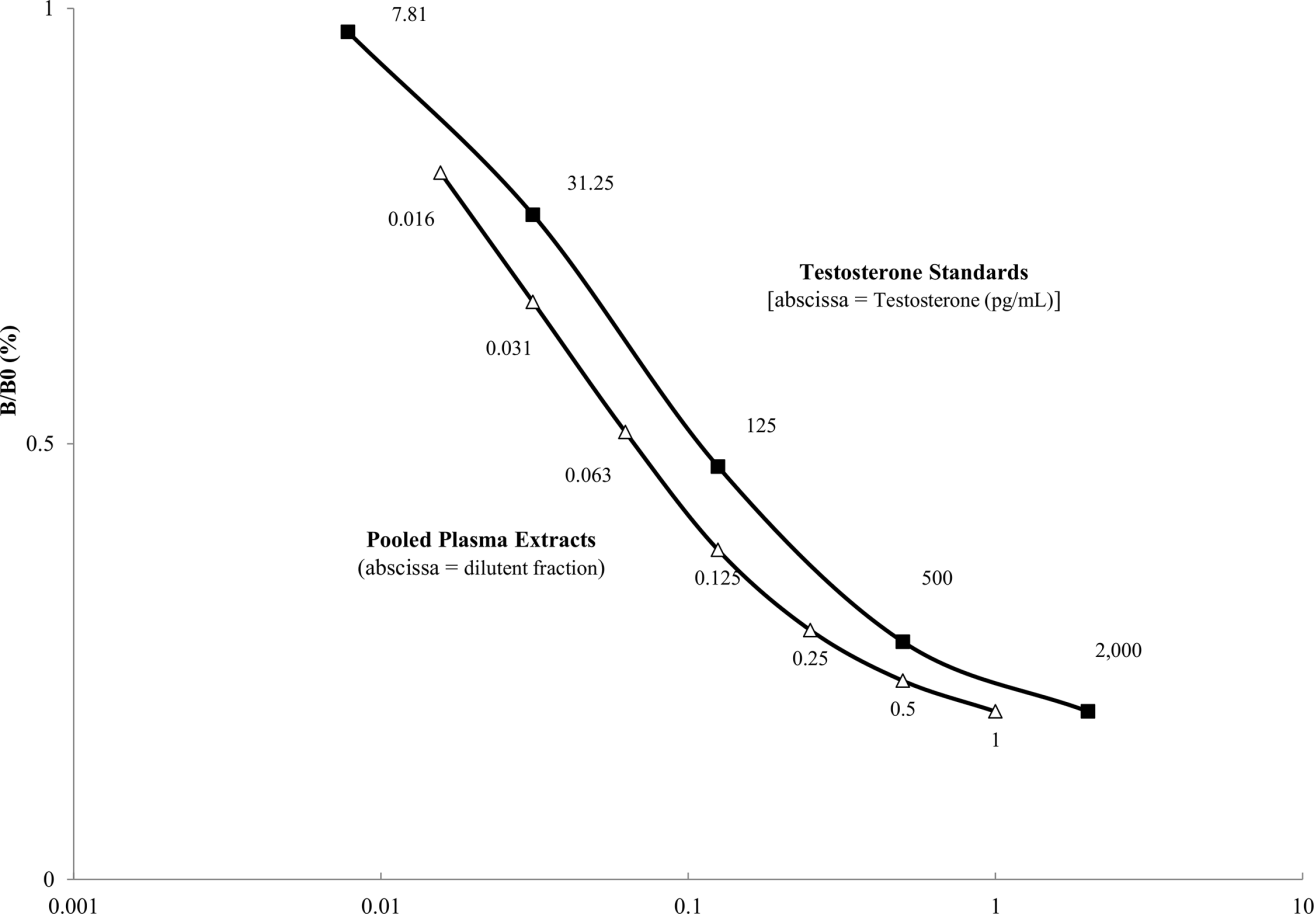
Results from a linearity assessment of a testosterone enzyme-linked immunosorbent assay (ELISA) using green sea turtle plasma extracts (six immature males). Serial dilutions of a pool of extracted green turtle plasma samples (hollow triangles) show parallelism with serial dilutions of testosterone standards (filled squares). Linearity suggests that the assay is measuring the same antigens in the plasma extracts as in the testosterone standards and, therefore, that the ELISA is valid for use with green sea turtle plasma extracts. % B/B_0_ = % bound/maximum bound

Mean extraction efficiency was 90.7% and the mean intra- and inter-assay coefficients of variation were 6.7% and 17.4%, respectively (n = 11 assays). We confirmed that the T ELISA measured the same antigen in the standard controls and plasma extracts because the slopes of curves from the known standard controls and serial dilutions of pooled plasma extracts demonstrated parallelism (Fig 2). We found no significant difference (r^2^ = 0.9962, p = 0.43; S2 Fig) in expected and observed concentrations when pooled plasma extracts were spiked with standard solutions; a finding consistent with little or no evidence of matrix interference.

### ELISA and RIA Results Comparison

**Fig 3:**
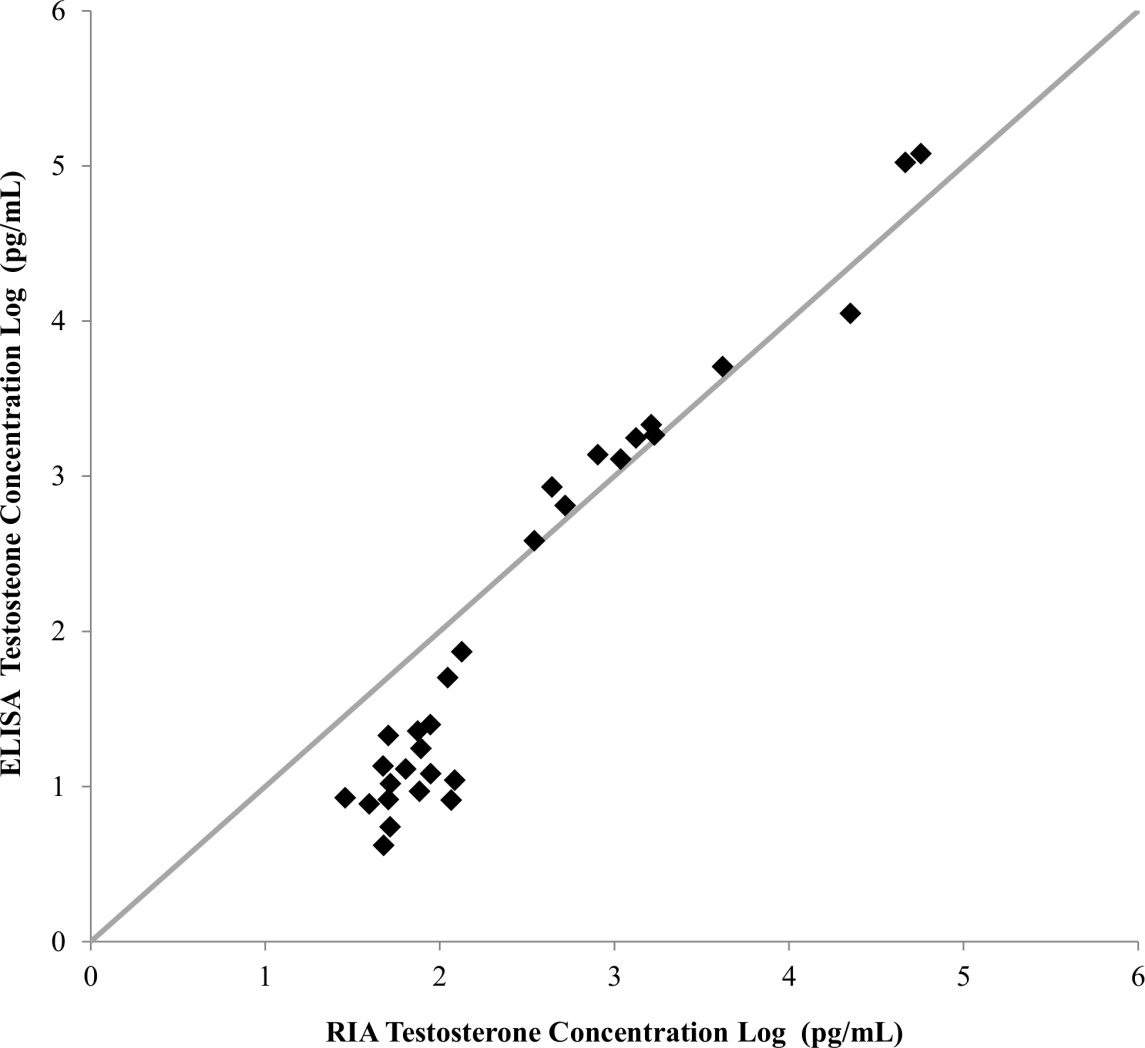
A correlation analysis between radioimmunoassay (RIA) and enzyme-linked immunosorbent assay (ELISA) testosterone concentrations of duplicate green sea turtle plasma samples. The high correlation coefficient (R = 0.97) indicates that the ELISA method is as a comparable method to RIA for determining immature green sea turtle sex. Note: the reference line (0 intercept and slope = 1.0) is illustrative of a perfect correlation

There was excellent correspondence (R = 0.97, Fig 3) between RIA- and ELISA-derived T concentrations for the samples that were analyzed by both methods which used different antibodies. Additionally, sex determination based on ELISA-derived T concentrations agreed with the sex determined via laparoscopy for all immature turtles. However, occurrence of high T values in some reproductively active females may lead to incorrect predictions of sex in both the RIA and ELISA assays. For example, one adult female captured along a migratory corridor in Panama had high T values (RIA: 1,333.0 pg/mL and ELISA: 1760.2 pg/mL) and was observed via laparoscopy to have shelled eggs. This turtle was incorrectly predicted to be a male in both assay techniques (RIA and ELISA). Testosterone is involved in the synthesis of female hormones (e.g. estradiol) and it is known that high T values in reproductively active female green turtles are typical (coinciding with migration, mating, and nesting activity [24,69]) and, therefore, can make it impossible to distinguish between reproductively active females and immature/mature males. However, this poses a problem for foraging aggregations only if reproductively active females are present.

### ELISA Measurements of San Diego Bay Turtles

**Fig 4:**
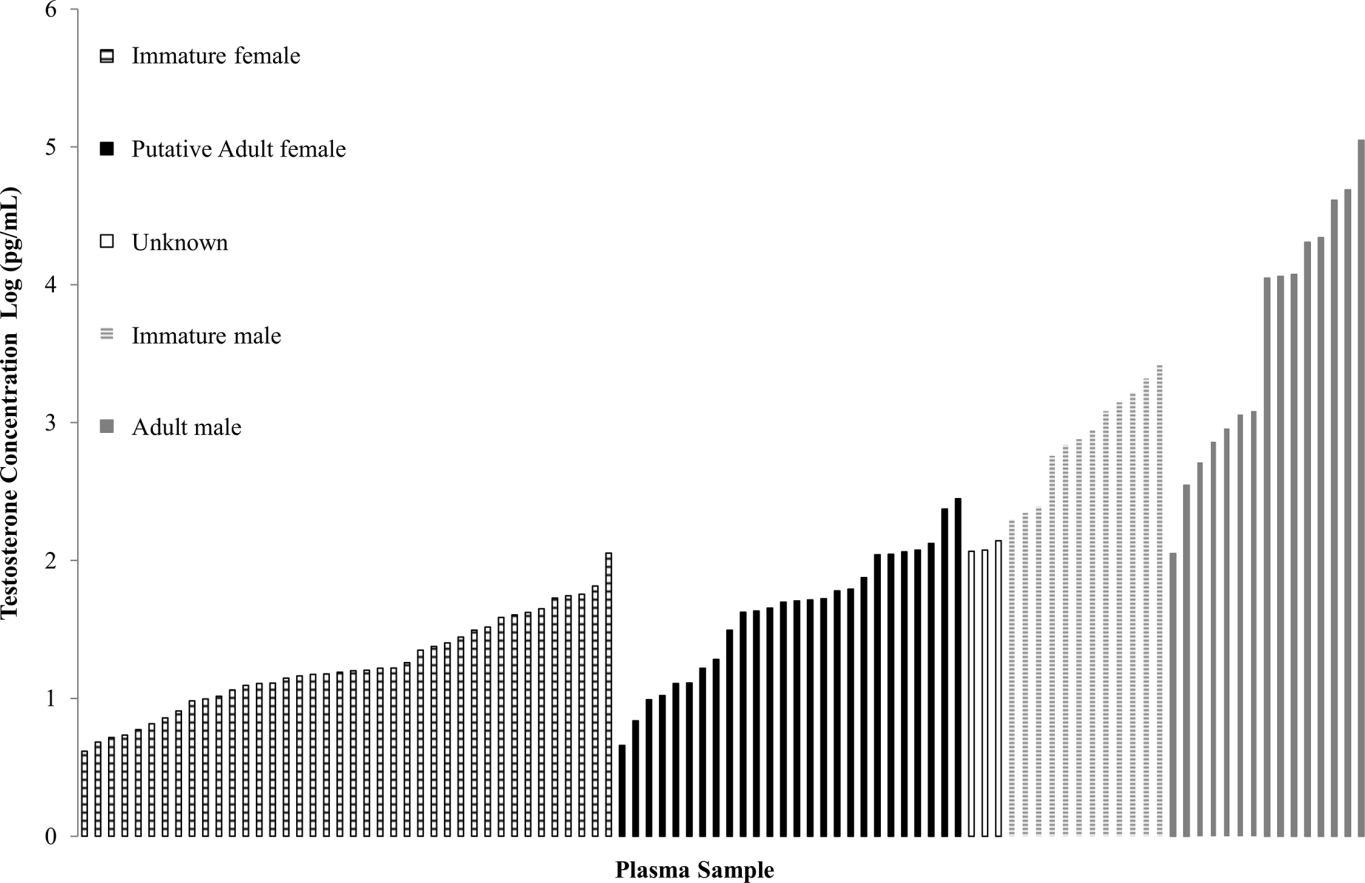
Mean testosterone concentration (pg/mL) of plasma samples (n = 96) collected from immature and putative adult green sea turtles (n = 69) captured in San Diego Bay, CA. Maturity was based on straight carapace length (> 90 cm) and sex was determined by testosterone levels and validated where possible by tail length, turtle size, and testosterone concentration upon future captures of the same individuals. Note: some turtles were recaptured and are represented by multiple data points

The range of T concentrations for all turtles in SDB was 4.15–112,094.2 pg/mL (Fig 4, S2 Table). The plasma samples of adult and immature male green turtles, in general, had higher T concentrations (112.4–112,094.2 pg/mL) than immature females and putative adult females (4.1–281.2 pg/mL, Table 1, Fig 4). However, they had overlapping values in the range of 112.4–281.2 pg/ml. Similar to Bolten et al. [51] we identified a T concentration range (‘unknown’ range) where the sex could not be determined for immature turtles because T concentrations did not fall within the T concentration ranges for either sex, but rather fell in a range in between immature male and female ranges. Of the 45 immature turtles (55 total plasma samples) examined in this study the female range was 4.1–113.1 pg/mL and the male range was 198.4–2,613.0 pg/mL. Three immature turtles had T concentrations (116.4, 119.0, 139.1 pg/mL) that fell within the unknown range of 113.1–198.4 pg/mL (Table 1, Fig 4).

**Table 1:** Mean ± standard error of mean (SEM) and (range) testosterone concentrations (pg/mL) of plasma samples (n = 96) obtained from different maturity states of female and male green turtles (n = 69) that forage in San Diego Bay, CA. Note: multiple blood samples were collected from 19 turtles captured on several occasions and, therefore, the results of those additional samplings are integrated within the maturity state at the time of capture based on size [straight carapace length (SCL)].

	Adult		Immature		
	(> 90 cm SCL)		(< 90 cm SCL)		
	Putative Females	Males	Females	Males	Unknown
	n = 21	n = 9	n = 32	n = 10	n = 3
Testosterone	67.8 ± 13.4	18,939.0 ± 7,737.3	23.9 ± 3.4	1,046.7 ± 223.1	124.9 ± 7.2
Concentration	(4.6–281.2)	(112.4–112,094.2)	(4.1–113.1)	(198.4–2,613.0)	(116.4–139.1)
Plasma Samples	n = 26	n = 15	n = 40	n = 12	n = 3

#### Estimating Probability of Sex Assignment

**Fig 5:**
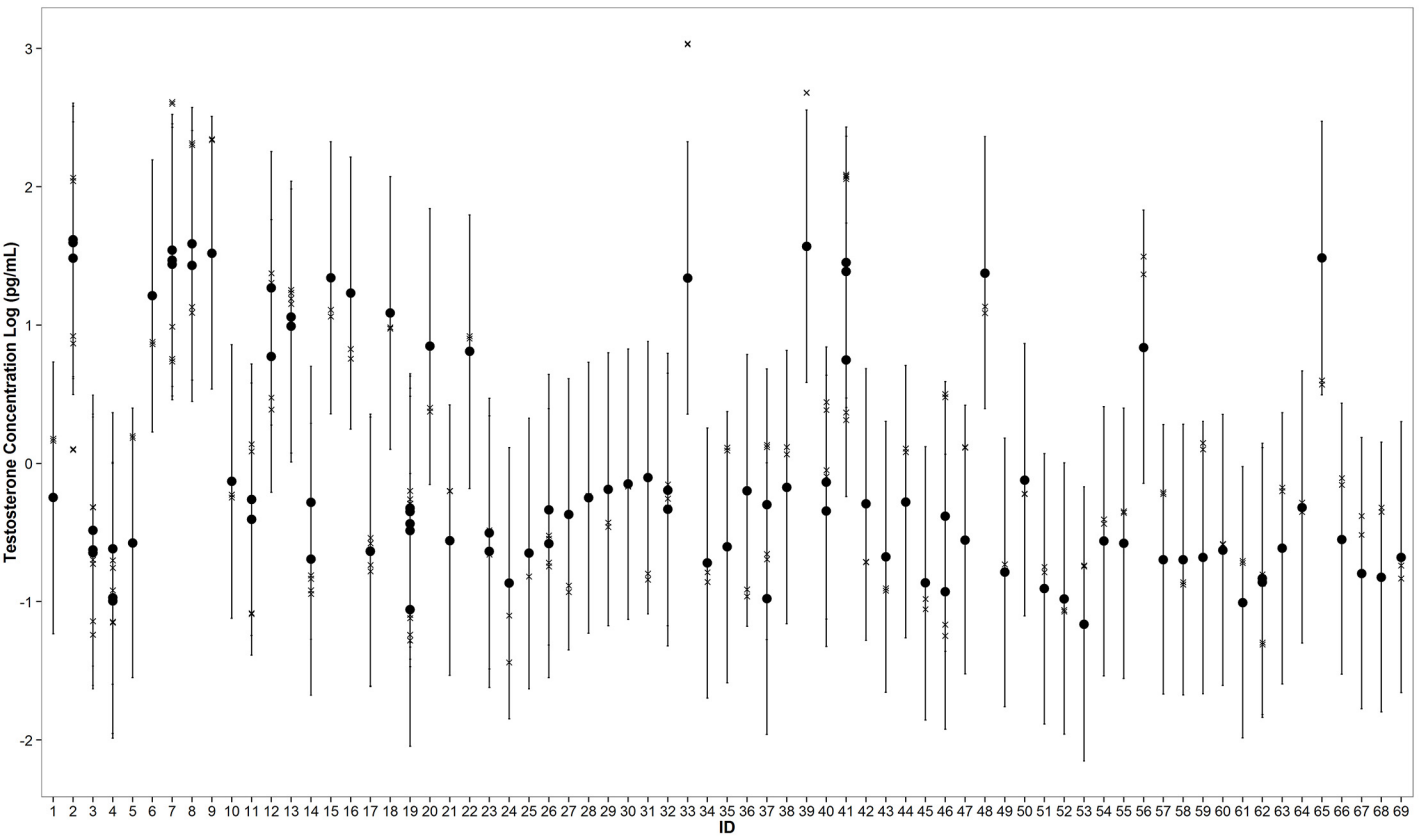
Posterior simulations of mean testosterone concentrations (in the natural logarithm space) for the 96 green sea turtle plasma samples used in this study. Filled circles indicate medians, vertical lines indicate 95% confidence intervals, and X symbols indicate observed values. Note: some turtles were recaptured and are represented by multiple data points. Three adult male turtles (IDs 7, 33, 39) have values outside of the 95% confidence intervals.

Convergence was reached in the MCMC sampling for all models (Gelman-Rubin Rhat statistic = 1.0). DIC indicated that Sex+SCL+DOY and Sex+SCL were the best two models for predicting sex probability; the difference in DIC between these models was 1.32. We used the best model (Sex+SCL+DOY) for parameter inference and this model was determined to be appropriate based on the results of posterior predictive simulations. The majority of observed concentrations (except 3 plasma samples collected from adult male turtles) were within 95% confidence intervals of simulated data (Fig 5) for each individual.

**Fig 6:**
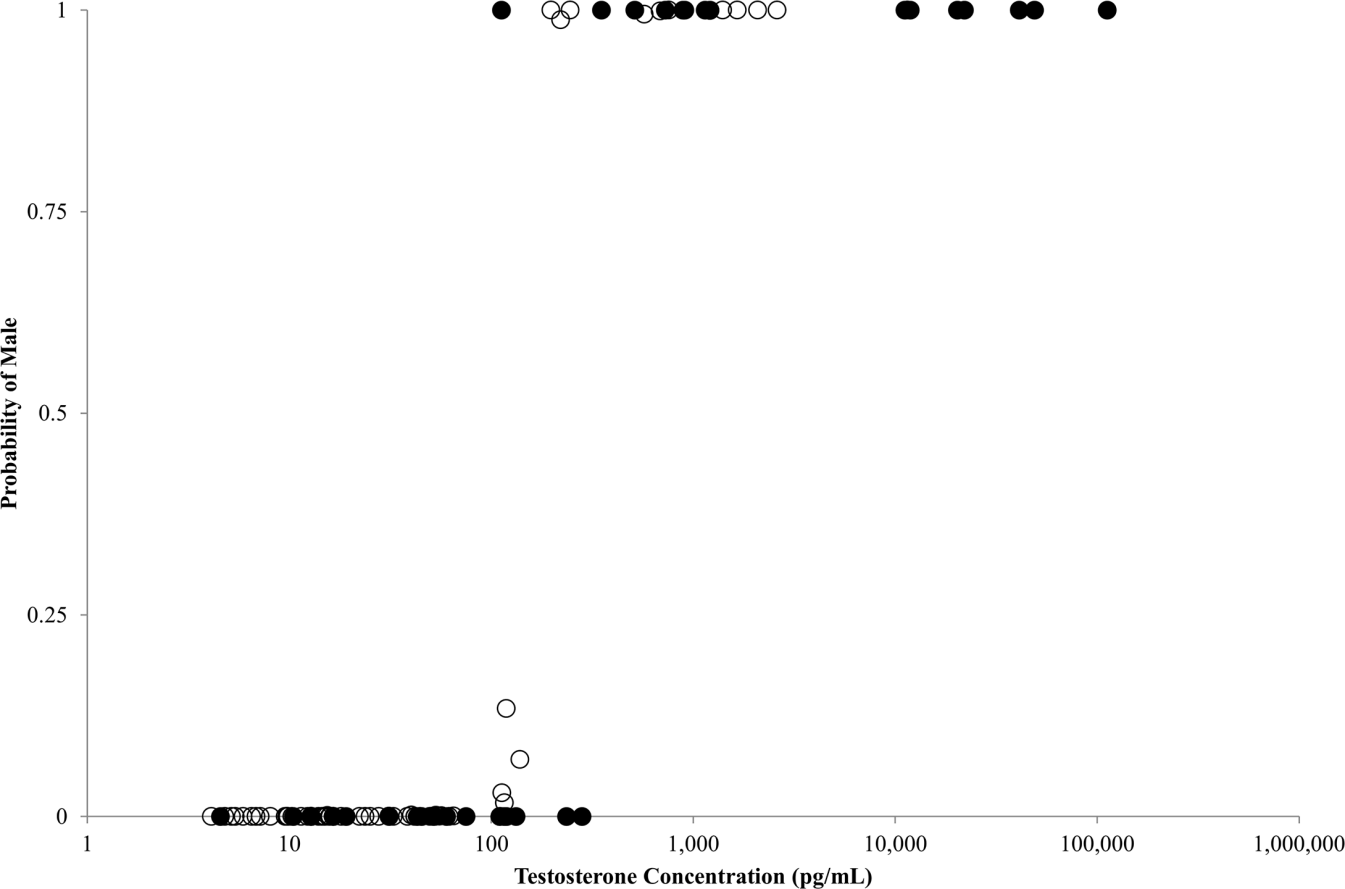
Results of a Bayesian statistical model to determine the probabilities of sex for 45 immature (hollow circles) and 24 putative adult (filled circles) green sea turtles in San Diego Bay, CA based on mean testosterone concentration (pg/mL). Note: some (n = 19) turtles were recaptured and are represented by multiple data points.

**Fig 7:**
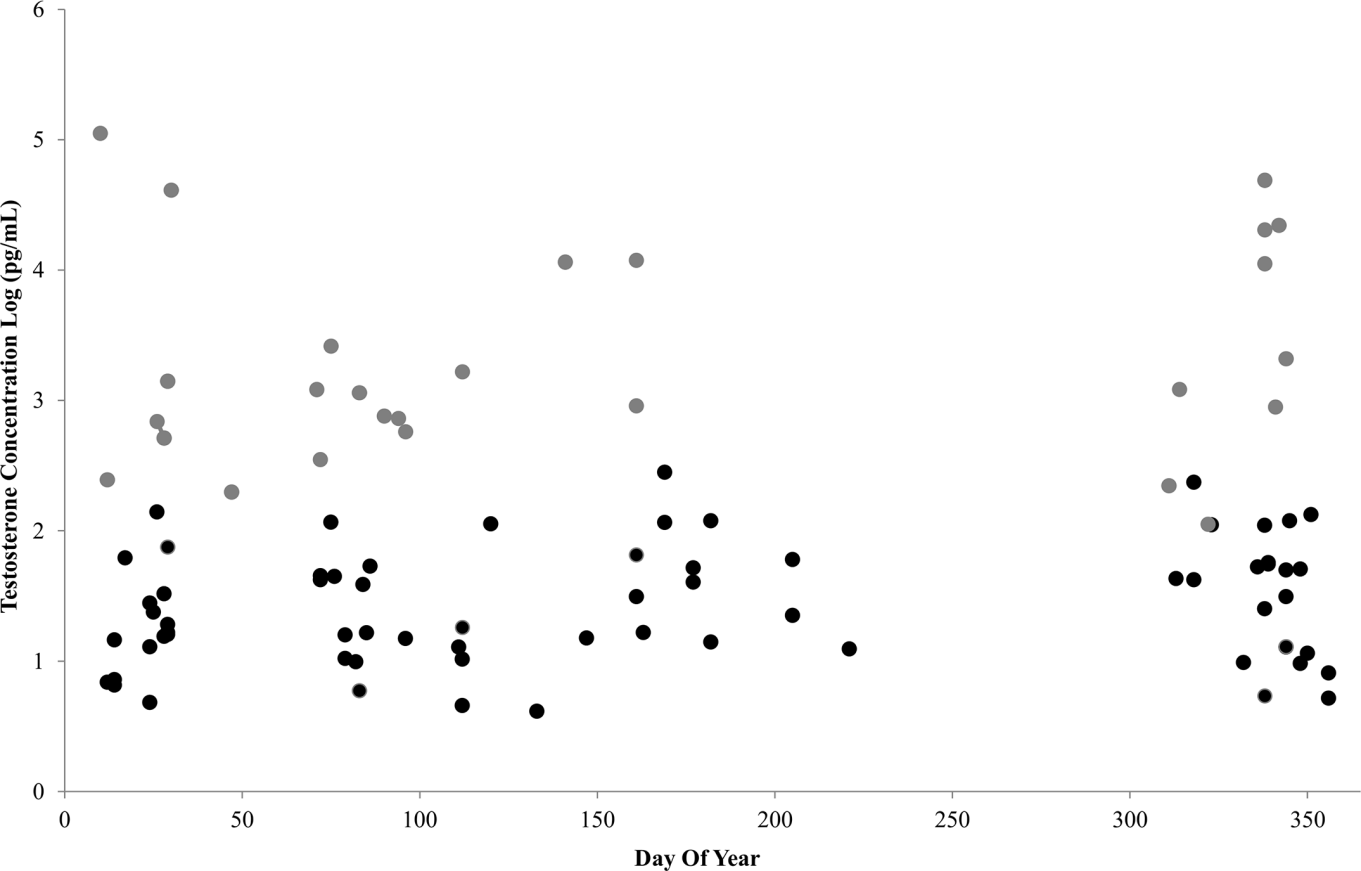
Bivariate analysis to examine if the date (day of year) of plasma sample collection influences log transformed mean testosterone concentrations (pg/mL) obtained from putative immature and adult green sea turtles which forage in San Diego Bay, CA. Grey circles represent plasma samples collected from male turtles and black circles represent female turtles. No strong trend was found.

All turtles were given probabilities of being male at < 0.14 or > 0.98 (Fig 6). One turtle (ID 2) was a known adult male (SCL = 99.6 cm) yet indicated a low average T concentration during winter (112.4 pg/mL, November 1998). Subsequent captures of this turtle over 11 years, however, resulted in high average T concentrations (11,184.2 and 728.5 pg/mL). These results indicated that T concentrations can vary within individuals or perhaps when water temperatures are cooler during winter. Additionally, sex may be difficult to assign, based solely on observed T, for green turtles with concentrations between 100 and 300 pg/mL. With sufficient samples, however, the model could predict sex reliably. We used a bivariate analysis (Fig 7) to examine if DOY influences T concentration and found no strong trend.

Treating all turtles with probability of male > 0.5 to be males, the sex ratio for the SDB foraging ground was estimated to be 2.83F:1M (51 females and 18 males), whereas samples obtained from putative immature turtles (< 90 cm SCL) had a 3.5F:1M (35 females and 10 males) sex ratio.

## Discussion

We used a commercially available ELISA T assay to provide the first estimate of sex ratio of a wild aggregation of green sea turtles at a foraging ground in the eastern Pacific Ocean. In addition to this field application, we developed a Bayesian approach to determine probability of sex from T measurements of individuals of known and unknown sex. Taken together, these study components will advance the practicability of sea turtle hormonal studies and allow for a broader understanding of sea turtle sex ratio in the eastern Pacific Ocean and globally.

### ELISA validation

Similar to Cocci et al. [27], we have demonstrated that enzyme immunoassay is a reliable and convenient tool for the rapid assessment of immature sea turtle sex, and therefore, sex ratio. The only limitation we encountered was that the assay was not reliable for predicting the sex of reproductively active females. This has also proven to be the case for RIA assays (Owens pers. comm.), and in both cases it is important to be able to identify and exclude these animals from the analysis. Sex determination via hormone assay in sea turtles is designed for immature turtles and sex assignment for larger turtles will always need to be accompanied by additional information (e.g. size, tail length, and water temperature) to confidently make a determination.

Our assay precision was similar to other laboratories that follow the same extraction procedure. For example, extraction efficiency ranges of 73.0–97.1%, intra-assay variation ranges of 4.9–13.3%, and inter-assay variation ranges of 13.8–23.9% [29,54,69–71] have been observed in previous studies employing RIA. For sea turtle hormone studies we suggest using the ambitious extraction method described in this manuscript because it is a superior methodology over extraction techniques provided in ELISA manufacturer protocols and it produces repeatable and dependable results.

We obtained corresponding T concentrations of duplicate samples using two T antibodies (RIA and ELISA) that had quite different cross-reactivity profiles; this is an encouraging result and suggests that, once validated, many other T assays might also work well in this species. Validation of the commercially available ELISA kits for use in sea turtle endocrinology studies is advantageous for researchers worldwide that do not have laboratory access to perform RIA assays, which require radioactive material licenses. Interestingly, the T ELISA used in this study may be of particular application for wildlife studies because the antibody used in the ELISA is a general androgen antibody (based on crossreactivities), which could be used for other matrices where androgens may be metabolized or degraded (e.g. feces). However, due to a break-down in the correspondence between the ELISA and the RIA at lower T concentrations where the RIA detected 2x more T than the ELISA, we caution that absolute hormone concentrations should not be compared between laboratories; nonetheless, the T concentrations of concern in this low range are below the threshold for determining sex.

The validation of the T ELISA for sea turtles will substantially broaden the application of sex ratio analyses for multiple species in many foraging areas worldwide that consist of cohorts of different age and maturity states and likely represent multiple genetic sources. Similar to an endocrinology study of East Pacific green turtles on the relationship of hormones and physiologic/environmental factors [35], the T ELISA (in addition to other biochemical and phenotypic markers) could also inform maturity or senescence and provide an indication of reproductive status of individuals within a population [72], one of several key demographic missing links for the SDB and other foraging populations [37]. In the future, after sufficient validation the ELISA technology could also be applied to other relevant hormones in sea turtles (e.g. corticosterone to examine stress response due to bycatch or stranding).

### ELISA Measurements of San Diego Bay Turtles

Assuming that the sex ratio was constant over time, the SDB foraging population on the whole is female biased. Available data from past and current studies of all life stages indicate sea turtle sex ratios are female-biased at most sites (Table 2). However, compared to other foraging locations globally, SDB has a more highly female-skewed total population and immature sex ratio (3:5F:1M; Table 2). Comparison of the present study to previous studies of sex ratios of immature green turtles at foraging grounds worldwide (Table 2) found nearly no bias (Hawaii and Bahamas) or moderately to heavily female-biased populations (Australia and Malaysia).

**Table 2:** Sex ratios of different life stages (hatchling, immature, and adult) of green turtles at various locations worldwide. Hatchling sex ratios are from nesting beaches while immature and adult sex ratios are from foraging grounds. NA: not applicable. CCL: curved carapace length. *: 1,199 turtles sexed, but number of turtles for each life stage was not provided.

Life Stage	Sexing Method	Location	Proportion of Females (%)	Sex Ratio (F:M)	Number of Turtles	Source
*Hatchling*	Incubation duration	Sri Lanka	57.2%		NA	[73]
	Nest temperature & gonad histology	Taiwan	84%		215	[74]
	Nest temperature	Heron Island, Australia	94%		NA	[75]
	Incubation duration	Alagadi Beach, Northern Cyprus	86–96%		NA	[76]
	Nest temperature & metabolic heating	Ascension Island	52.5–99.8%		NA	[77]
*Immature*	Blood hormones	Hawaii		1.0:0.96	66	[71]
	Laparoscopy	Shoalwater Bay, Australia		1.74:1.0	738 (< 65.1 cm CCL)	[78]
	Blood Hormones	Inagua, Bahamas		1.4:1.0	111	[51]
	Laparoscopy and blood hormones	Heron Island, Australia		2.0:1.0	200	[20]
	Laparoscopy	Clack Reef, Australia		2.2:1.0	* (> 65.1 cm CCL)	[79]
	Laparoscopy	Shoalwater Bay, Australia		3.26:1.0	637 (> 65.1 cm CCL)	[78]
	Blood hormones & mark-recapture	San Diego, California		3.5:1.0	45	Present study
	Laparoscopy	Sabah, Malaysia		4.0:1.0	75	[80]
	Laparoscopy	Clack Reef, Australia		4.2:1.0	* (< 65.1 cm CCL)	[79]
*Adult*	Tail length	Gulf of Carpentaria, Australia	2.0%		42	[81]
	Tail length	Masirah Island, Sultanate of Oman	47%		242	[82]
	Laparoscopy	Shoalwater Bay, Australia		1.78:1.0	620	[78]
	Laparoscopy	Clack Reef, Australia		2.1:1.0	*	[79]
	Blood hormones & mark-recapture	San Diego, California		2.83:1.0	30	Present study

It is unlikely that reproductively active females with high T concentrations confounded our results significantly as all turtles considered immature or adult males based on high T concentration were re-captured with distinguishable male-sized tails. However, two immature turtles (considered to be male based on T) were caught a single time and measured 85.9 cm SCL (ID 6) and 59.3 cm SCL (ID 56) with T concentrations of 688.7 pg/mL and 2613.0, respectively. It is unlikely that either of these were mature female turtles becoming reproductively active prior to the onset of the peak breeding season (Michoacán and Revillagigedo Archipelago: October to November [49,50]) and migration to Mexican nesting grounds because they were sampled in November and March, respectively. Nonetheless, if these two immature male turtles were indeed reproductively active female turtles it would increase the female-biased sex ratio of immature turtles in the SDB aggregation to 3.7F:1.0M.

Female biases in sea turtle foraging populations may be driven by a number of factors. The most likely explanation for a female-biased foraging ground is female-biased hatchling sex ratios because foraging grounds represent a concentration over many years of the sex ratio of the rookeries from which the turtles were hatched [15]. However, estimating sex ratios in hatchlings remains difficult (or not possible) without sacrificing the animal, so using the T ELISA to estimate sex ratios in immature turtles at foraging grounds is an effective method to detect changes or signs of skewed ratios. Also, coupled with genetic mixed-stock analyses, the sex ratio on foraging grounds can be used to estimate the sex ratio at source rookeries [83]. Although only small volumes of plasma (≤ 50 μL) can be collected from hatchlings due to their size, ELISA sensitivity should be sufficient to determine hatchling sex and may provide a less-invasive approach for determining hatchling sex ratios. A potentially critical result is the facilitation of studies to identify the degree to which climate change may impact sex ratios of annual hatchling cohorts (if applied over multiple nesting seasons).

Another possible explanation for a female-bias at foraging grounds is differences in habitat preference between males and females. Previous research in other reptiles (hatchling crocodiles and juvenile snapping turtles) has found that thermal behavior was influenced by incubation temperature [84,85]. For example, hatchling crocodiles incubated at higher temperatures were introduced into thermal gradients and they selected a gradient which maintained a higher body temperature compared to their counterparts incubated at a lower temperature. Both studies suggested that temperature selection may influence thermal habitat choice and it was possibly due directly or indirectly to incubation temperature, and thus, the animals’ sex. Because the water temperature in the SDB was artificially increased due to the power plant effluent near our capture location, it is possible more females were captured due to their selection of warmer water temperatures (or other covariates). Therefore, male turtles may forage at different locations within the SDB (or at other foraging sites in the East Pacific not yet identified) associated with lower water temperatures. Nonetheless, we believe the plasma samples used for this study provided a representative sample for the SDB population, and further investigation is required to support sex differences in habitat preference for sea turtles.

An alternative explanation for the female-biased SDB population is that female turtles exhibit different migratory periodicity than males. In Australia, the remigration interval to breeding grounds for adult female green turtles (5.8 years, [86]) is much longer than for males (2.08 years, [87]); therefore, there may be proportionately fewer males available for sampling in the foraging aggregation because they are instead at the breeding grounds. While the remigration interval for adult male green turtles in the East Pacific is unknown, females migrate to Mexican nesting grounds (where SDB turtles have been tracked by satellite telemetry, [62]) every 1.8 to 3.0 years [88,89]. Perhaps females in SDB forego migration in some years while males may migrate to nesting beaches more frequently, thereby leaving a greater number of females in the bay on average. However, this rationalization is unlikely because of the high female bias in the SDB immature turtles that do not migrate for mating.

#### Estimating Probability of Sex

A novel outcome of this study was a statistical model that provides probability of sex for turtles that exhibit intermediate T concentration. The model requires data on T concentrations from individuals of known sex. Other covariates, such as size, tail lengths, and a seasonal index (e.g. day of year), may be useful in providing precise estimates. In our analyses, however, tail lengths were not useful—perhaps because of missing data in some individuals or because an alternative tail measurement parameter might be more suitable for determining sex in the SDB population, similar to findings for loggerhead sea turtles [90]. The probabilistic outcome is helpful in estimating a sex ratio because there is no need to establish arbitrary thresholds for T concentrations. Previous studies that determined sex ratio using RIA only (without validation by laparoscopy) still reported immature animals of unknown sex due to plasma T concentrations falling within the unknown range (e.g., [51,71]). Conversely, the statistical model can provide the probability of sex for immature turtles. Moreover, the model can be applied to other populations and species, thereby enhancing baseline information on sex ratios to inform management decisions for conserving endangered sea turtles.

We assumed normal distributions for the observed standardized and log-transformed ELISA T concentrations and believe this was a reasonable approximation of the data. The mean of the normal distribution was treated as a linear function of covariates, which may be improved by including products and other functions of covariates if sufficient samples are collected from each individual. In our data, repeated samples from the same individuals were not collected within a year to justify inclusion of more complex functions in the model. Investigation into possible effects of collection date upon T concentration did not show a strong pattern (Fig 7), suggesting that there was no seasonal effect, however, to determine possible seasonal fluctuations in T titers, it is best to repeatedly sample the same individuals within a year [28].

### Conservation implications

Female-biases in breeding populations may be beneficial for species recovery due to an increase in the number of breeding females, and therefore, population growth potential [91]. For example, a female bias may explain the population increase of the threatened Pacific Mexico green turtle population [48] since it was protected in 1978 [92].

Climate change scenarios indicate that the problem of near complete feminization for certain rookeries of different sea turtle species could occur within the next ten to fifteen years [93,94] or longer (by 2070, [19]; 159 years, [91]) without phenomenological shifts or other behavioral adaptations. Current sex ratio baseline information will be informative for predicting climate warming conservation concerns for sea turtles, and sex ratio information for each sea turtle species is vital for inferring population status and the survivorship of each sex. Indeed, Labrada-Martagón et al. [72] emphasized how physiological approaches (hormone determinations) can provide valuable demographic information for marine vertebrate conservation. Knowledge of population sex ratios and their inclusion in management plans will create a more comprehensive approach to the conservation of sea turtles globally.

## Supporting Information

S1 Fig Example of a testosterone ELISA plate layout sheet used in our laboratory. (PDF)

S2 Fig Linear regression analysis of a matrix-interference test to examine if there was any potential interference caused by substances within sea turtle plasma samples. We found no significant difference (r^2^ = 0.9962, p = 0.43) in expected and observed testosterone concentrations when pooled green sea turtle plasma extracts were spiked with standard solutions; a finding consistent with little or no evidence of matrix interference.
(TIF)

S1 Table Morphometrics and testosterone concentration of plasma samples collected from 30 green sea turtles captured in Panama and Bermuda. F: female and M: male.
(XLSX)

S2 Table Morphometrics and testosterone concentration of plasma samples collected from 69 green sea turtles captured in San Diego Bay, California. Note: some turtles were recaptured multiple times. F: female, M: male, and U: unknown sex.
(XLSX)
