# Supplementary material for: First Assessment of the Sex Ratio for an East Pacific Green Sea Turtle Foraging Aggregation: Validation and Application of a Testosterone ELISA
Source: PLoS One. 2015 Oct 14;10(10):e0138861. doi: 10.1371/journal.pone.0138861 (PMC4605721; doi:10.1371/journal.pone.0138861)
Supplement: S1 Fig — (PDF) [file pone.0138861.s001.pdf]

ENZO Testosterone Plate Layout

**A1** Blank	**A2** Std 5	**A3**	**A4**	**A5**	**A6**	**A7**	**A8**	**A9**	**A10**	**A11**	**A12** Std 4
**B1** TA	**B2** Std 6	**B3**	**B4**	**B5**	**B6**	**B7**	**B8**	**B9**	**B10**	**B11**	**B12** Std 3
**C1** NSB	**C2** Std 7	**C3**	**C4**	**C5**	**C6**	**C7**	**C8**	**C9**	**C10**	**C11** Low Control	**C12** Std 2
**D1** Bo	**D2** Low Control	**D3**	**D4**	**D5**	**D6**	**D7**	**D8**	**D9**	**D10**	**D11** Medium Control	**D12** Std 1
**E1** Std 1	**E2** Medium Control	**E3**	**E4**	**E5**	**E6**	**E7**	**E8**	**E9**	**E10**	**E11** High Control	**E12** Bo
**F1** Std 2	**F2** High Control	**F3**	**F4**	**F5**	**F6**	**F7**	**F8**	**F9**	**F10**	**F11** Std 7	**F12** NSB
**G1** Std 3	**G2**	**G3**	**G4**	**G5**	**G6**	**G7**	**G8**	**G9**	**G10**	**G11** Std 6	**G12** TA
**H1** Std 4	**H2**	**H3**	**H4**	**H5**	**H6**	**H7**	**H8**	**H9**	**H10**	**H11** Std 5	**H12** Blank

Kit Lot #:_________________________ Exp. Date:_____________________ Date Run:___________________ Tech:____________________________

1st Incubation: Start time:_____________

2nd Incubation: Start time:_____________

End time:_____________

3rd Incubation: Start time:_____________

End time:_____________

Notes:____

____

____

____

____

Pipette Calib 100 uL mili-Q H_2O =______ g Pipette: ____

Pipette Calib 40 uL mili-Q H_2O = ______ g Pipette: ____
