# Supplementary figures and images for: First Assessment of the Sex Ratio for an East Pacific Green Sea Turtle Foraging Aggregation: Validation and Application of a Testosterone ELISA
Source: PLoS One. 2015 Oct 14;10(10):e0138861. doi: 10.1371/journal.pone.0138861 (PMC4605721; doi:10.1371/journal.pone.0138861)

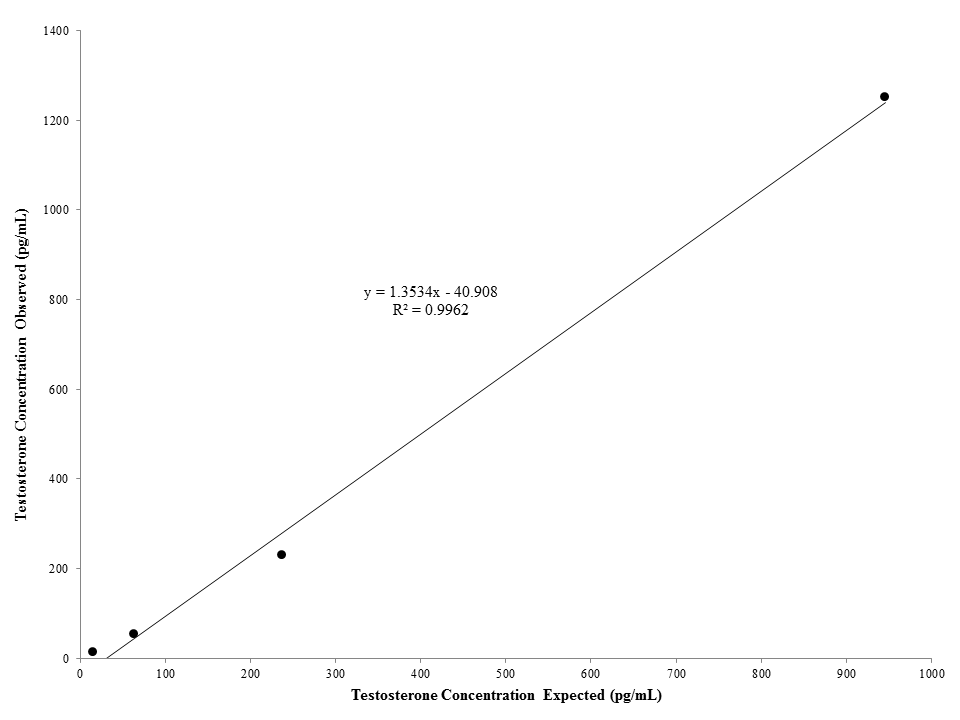

Supplement: S2 Fig — We found no significant difference (r2 = 0.9962, p = 0.43) in expected and observed testosterone concentrations when pooled green sea turtle plasma extracts were spiked with standard solutions; a finding consistent with little or no evidence of matrix interference. (TIF) [file pone.0138861.s002.tif]
